# Extracranial radiosurgery with volumetric modulated arc therapy: Feasibility evaluation of a phase I trial

**DOI:** 10.3892/ol.2013.1276

**Published:** 2013-03-29

**Authors:** FRANCESCO DEODATO, SAVINO CILLA, GABRIELLA MACCHIA, LUCIANA CARAVATTA, SAMANTHA MIGNOGNA, MARIANGELA MASSACCESI, VINCENZO PICARDI, CINZIA DIGESU, GIUSEPPINA SALLUSTIO, PIERLUIGI BONOMO, ANGELO PIERMATTEI, GABRIELLA FERRANDINA, GIOVANNI SCAMBIA, VINCENZO VALENTINI, NUMA CELLINI, ALESSIO G MORGANTI

**Affiliations:** 1 Units of Radiotherapy, Fondazione di Ricerca e Cura “Giovanni Paolo II”, Università Cattolica del S. Cuore;; 2 Medical Physics, Fondazione di Ricerca e Cura “Giovanni Paolo II”, Università Cattolica del S. Cuore;; 3 Palliative Therapies, Fondazione di Ricerca e Cura “Giovanni Paolo II”, Università Cattolica del S. Cuore;; 4 Radiology, Fondazione di Ricerca e Cura “Giovanni Paolo II”, Università Cattolica del S. Cuore;; 6 Gynaecologic Oncology, Fondazione di Ricerca e Cura “Giovanni Paolo II”, Università Cattolica del S. Cuore;; 5 Departments of Medical Physics, Università Cattolica del S. Cuore, I-86100 Campobasso, Italy; 7 Gynaecologic Oncology, Università Cattolica del S. Cuore, I-86100 Campobasso, Italy; 8 Radiotherapy, Università Cattolica del S. Cuore, I-86100 Campobasso, Italy

**Keywords:** extracranial radiosurgery, stereotactic body radiosurgery, volumetric modulated arc therapy, volumetric intensity modulated arc therapy, feasibility, phase I

## Abstract

The aim of this study was to report early clinical experience in stereotactic body radiosurgery (SBRS) delivered using volumetric intensity modulated arc therapy (VMAT) in patients with primary or metastatic tumors in various extra-cranial body sites. Each enrolled subject was included in a different phase I study arm, depending on the tumor site and the disease stage (lung, liver, bone, metastatic), and sequentially assigned to a particular dose level. Technical feasibility and dosimetric results were investigated. The acute toxicity, tumor response and early local control were also studied. In total, 25 lesions in 20 consecutive patients (male/female, 11/9; median age, 67 years; age range, 47–86 years) were treated. Of these 25 lesions, 4 were primary or metastatic lung tumors, 6 were liver metastases, 8 were bone metastases and 7 were nodal metastases. The dose-volume constraints for organs at risk (OARs) were observed in 19 patients using a single-arc technique. Only in one patient were two arcs required. The treatment was performed without interruption or any other technical issues. The prescribed dose ranged from 12–26 Gy to the planning target volume (PTV). Delivery time ranged from 4 min to 9 min and 13 sec (median, 6 min and 6 sec). No incidence of grade 2–4 acute toxicity was recorded. The overall response rate was 48% (95% confidence interval (CI), 24.2–70.2) based on computed tomography (CT)/magnetic resonance imaging (MRI) and 89% (95% CI, 58.6–98.7) based on the positron emission tomography (PET) scan. SBRS delivered by means of VMAT allowed the required target coverage to be achieved while remaining within the normal tissue dose-volume constraints in the 20 consecutive patients. VMAT-SBRS resulted in adequate technical feasibility; the maximum tolerable dose has not yet been reached in any study arm.

## Introduction

The term stereotactic body radiosurgery (SBRS) implies the delivery of a focused single dose of radiation therapy (1). This technique has been used in the treatment of various types of cancer in different anatomic sites, including primary or metastatic lung tumors (2–4), primary or secondary liver tumors (5–7), pancreatic tumors (8), gynecological cancer recurrences (9) and bone metastases (10).

With the delivery of a very high dose single fraction of radiation therapy, SBRS requires steep dose gradients, usually obtained by dynamic techniques or non-coplanar fixed fields. SBRS also requires high precision in the treatment delivery process. Therefore, it requires a short fraction length to reduce the risk of intra-fraction set-up deviations or organ motion.

Volumetric modulated arc therapy (VMAT) is a novel radiotherapy technique. VMAT differs both from standard intensity-modulated radiation therapy (IMRT) and three-dimensional conformal radiotherapy (3D-CRT), which operate in static conditions, and is characterized by dose delivery by dynamic arcs (11). During VMAT, the delivery of radiation occurs with a rotational movement of the linear accelerator (LINAC) gantry while a continuous variation of the beam’s profile and intensity is obtained. VMAT requires a sophisticated technique for complex treatment planning. As VMAT has evolved from IMAT, VMAT has the advantage of high-dose conformity and improved sparing of healthy tissues. Therefore, VMAT may be theoretically useful for dose escalation and improved tumor control probability. In addition, the duration of dose delivery is very short, allowing the advantages of IMRT (high conformity index) to be combined in a reduced treatment time. The consequences are represented by a higher operating efficiency of each treatment unit, enhanced patient comfort and reduced risk of intrafraction deviations both in terms of set-up errors or organ motion.

For these reasons, VMAT is a potentially ideal technique for SBRS. However, it is not yet clear whether administration of very high doses in single fraction delivery with such a complex technique is possible. Additionally, the true capacity of VMAT to respect dose-volume constraints even in the case of high doses per fraction is uncertain. To the best of our knowledge, no data on VMAT-SBRS have been published.

Based on this background, a feasibility study regarding SBRS based on the VMAT technique (DESTROY-2 protocol) has been planned. The purpose of this analysis is to report the preliminary results of this study.

## Materials and methods

### Study characteristics

This trial was conceived as a prospective dose escalation study. All patients consecutively observed at our Radiotherapy Unit (Catholic University, Campobasso, Italy) and matching the inclusion criteria were enrolled. The trial was approved by the Catholic University Institutional Review Board. A preliminary evaluation of technical feasibility was planned following the enrollment of the first 20 patients. Written informed patient consent was obtained from the patients.

### Study objectives

The primary study end point was the definition of maximum tolerated dose (MTD) of SBRS with VMAT. The secondary objectives of the study were: i) feasibility evaluation in terms of dose-volume constraints; ii) analysis of the correlation between dosimetric and toxicity data; iii) analysis of the clinical response and iv) evaluation of local control.

### Radiosurgery dose escalation

Each enrolled subject was included in a study arm according to the tumor site and disease stage, as demonstrated in Table I. Patients were sequentially assigned to a specific dose level as detailed in Table II. VMAT dose escalation was based primarily on the acute and subacute toxicity, as late toxicity is capable of occurring months or years later. Acute-subacute toxicities were defined as those that occurred within 6 months of receiving treatment. Toxicities registered ≥6 months post-radiation were defined as late toxicities. Dose-limiting toxicities (DLTs) were defined as any treatment-related non hematological adverse effects rated as ≥grade 3 or any hematological toxicity rated as ≥grade 4, by the National Cancer Institute Common Terminology Criteria for Adverse Events v.4.03 (12). If the DLT was not observed in the three patients at a given dose level, the trial proceeded to the next dose level, provided that 6 months of follow-up had occurred following the VMAT for the third patient of the cohort. If a DLT occurred in one of the three patients at a given dose level, treatment of up to three additional patients at this dose level was required. If the DLT occurred in more than one patient of the three patients’ cohort, dose escalation was halted, and the dose level below that was considered to be the MTD. If a DLT occurred in two or more patients of the expanded six-patient cohort, dose escalation was terminated, and the dose level below that was considered to be the MTD. If a DLT occurred in less than two patients of the expanded six-patient cohort, the trial proceeded to the subsequent dose level. Different total VMAT doses were selected on initiation of the study as the highest dose levels to be evaluated, and were dependent on the study arm. Late toxicities were continuously monitored regardless of whether patients had documented disease progression.

### Inclusion criteria

The following inclusion criteria were used: histological diagnosis of solid tumor (with the exception of germinal tumors) with the site and tumor stage as demonstrated in Table I; age, >18 years; ECOG performance status, 0–3; adequate bone marrow function, which included neutrophil count, >1500 *μ*l; platelets, >100,000/ml; hemoglobin, >9 g/dl. Additionally, for patients receiving irradiation to the kidney (lumbar/abdominal area) the inclusion criterion was creatinine, <1.8 mg/dl; while the criteria for patients receiving irradiation to the liver were total bilirubin, <3 mg/dl; lactate dehydrogenase, <3-fold the normal value; aspartate aminotransferase, <3-fold the normal value; alanine aminotransferase, <3-fold the normal value and alkaline phosphatase, <3-fold the normal value. Previous treatment with surgery and/or chemotherapy and/or radiotherapy was permitted.

### Exclusion criteria

The following exclusion criteria were employed: ECOG, >3; the presence of medical conditions which contraindicate radiation therapy, such as connective system disorders, severe uncompensated heart disease (in case of heart irradiation), acute diverticulitis, ulcerative colitis and pelvic inflammatory disease (in case of irradiation of the pelvis); comorbidities that in the opinion of the referring physician may constitute a risk to clinical trial participation.

### End points and statistical analysis

Toxicity was evaluated by the Common Toxicity Criteria for Adverse Events (CTCAE) scale, version 4.03 (12). The presence of focal liver reaction was evaluated as outlined by Herfarth *et al*(13). The survival curves were calculated with the Kaplan-Meier method (14). Statistical analysis was performed using SYSTAT software, version 11.0 (SPSS, Inc.; Chicago, IL, USA).

### Patient set-up

Patient immobilization was performed with a stereotactic body frame (SBF; Elekta; Crawley, UK), which is an immobilization device used to define a stereotactic system of coordinates for the target position as opposed to anatomical landmarks such as bony structures or skin markers. This device was described in detail by Lax *et al*(15) and clinical results have been published by Blomgren *et al*(16). The SBF is a U-shaped rigid plastic frame, within which different sized vacuum pillows allow a reproducible immobilization for the repositioning of each patient. Patient repositioning is supported by a laser system directly attached to the body frame at defined longitudinal positions. Alignment of the stereotactic coordinate system of the SBF to the isocenter of the computed tomography (CT) machine or the treatment unit is performed by a stereotactic arc with scales in the anterior-posterior and lateral directions. The longitudinal stereotactic coordinate is found on a scale along the body frame sidewalls and is simply read on each CT-slice using a system of straight and oblique copper pieces, which function as fiducials. Moreover, to reduce the respiration mobility of targets close to the diaphragm, a compressor, attached to the SBF by a rigid arc, may be mechanically pressed into the patient’s epigastrium to decrease the respiration motion.

### CT simulation

To evaluate the reproducibility of the set-up, three CT scan evaluations were performed on three different days, with the aim of verifying that the set-up deviation was <3 mm. In order to evaluate the organ motion produced by the respiratory movements, target displacement was measured. During free breathing, 30 axial CT scans were performed on the same slice. In the case of a displacement >5 mm, the abdominal compressor of the SBF was applied and the CT scan for organ motion assessment was repeated. The final CT simulation, for the acquisition of axial images necessary for stereotactic localization and plan calculations, was produced with a spiral technique. Subsequently, 3-mm scans were acquired with a 3-mm interval between scans in the target region. For the remainder of the SBF, 10-mm slices were acquired and the interval between scans was 10 mm. In treating abdominal or pelvic targets, patients received 2 cc of oral Gastrografin, diluted in 0.5 l of water 30 min prior to CT scan acquisition. In case of mediastinal, abdominal or pelvic target volumes, intravenous infusion of an iodinated contrast medium was also used.

### Volumes of interest

The clinical target volume (CTV) was defined as the gross tumor volume (GTV) in case of metastases and primary lung tumors. A 5-mm margin was added to the GTV to define the CTV in primary tumors of the liver. The planning target volume (PTV) was individually defined for each patient based on the internal margin (IM) and the set-up margin (SM) assessment. The IM was defined based on respiratory excursions in 3D. The SM was set at 3 mm according to the ROSEL study (17). The OARs considered included: i) The thorax: the spinal cord, lungs, esophagus, heart, brachial plexus, peripheral nerves, large vessels, trachea and ribs; ii) The abdomen: the spinal cord, liver, stomach, small bowel, colon and kidneys; iii) The pelvis: the sacral plexus, small bowel, colon, rectum, anal canal, bladder, femoral heads and penile bulb.

### Prescription

A uniform method for the selection of the prescription isodose surface (IDS) was adopted. According to the ROSEL study (17), for each plan the IDS was selected as the greatest IDS fulfilling the two following criteria: 95% of the PTV volume reached 100% of the prescription dose and 99% of the PTV reached ≥90% of the prescription dose. The aim was to increase the dose heterogeneity so as to intensify the dose within the GTV. The maximum dose within the PTV should not exceed 140% of the prescribed dose. Careful attention was paid to ensure the maximum dose always remained within the GTV.

### Treatment planning

VMAT plans were generated using the ERGO^++^ treatment planning system (TPS), version 1.7.3 (Elekta). This is an anatomy-based TPS that supplies a simplified approach to creating VMAT plans, by predefining a series of aperture shapes using Boolean operations in conjunction with the beam’s eye view of the target and OARs. In the current study, all plans were generated with a single-arc rotation except for patient number 16, who was treated for two liver lesions and therefore required two arcs. The dose calculation was performed using the pencil beam algorithm with inhomogeneity correction and a dose grid resolution of 2 mm. VMAT plans were exported to the record and verify (R&V) system Mosaiq v. 1.6 (Impac Software; Elekta) by DICOM-RT for later irradiation. Table III lists the dose-volume constraints used (5,18,19).

### Quality assurance

Set-up deviation and organ motion assessments were performed as previously described. For quality assurance through treatment planning and delivery, two independent checks (IC1 and 2) were performed by medical and physics staff, as previously described (20).

### Supportive therapy

Supportive therapy was prescribed according to the irradiated site. In the case of irradiation of two anatomic sites, such as the chest and the abdomen, supportive care was provided for both sites. In patients receiving irradiation to the chest, prescriptions included betamethasone 0.5 mg orally, 3 times daily for 1 month, followed by a gradual reduction, associated with gastric protection (H2-inhibitors). Patients receiving abdominal irradiation were prescribed metoclopramide 10 mg orally, 3 times a day, for ≤1 week following radiation therapy and rabeprazole 40 mg orally, once daily for 12 months (in case of irradiation of the stomach and/or the duodenum only). In addition, patients receiving irradiation to the upper abdomen were prescribed dexamethasone 12 mg intravenously (IV) 1 fl immediately prior to radiosurgery and 6 h after treatment, while 3 mg granisetron was administered immediately prior to radiosurgery by IV slow infusion.

### Evaluation of response and follow-up

The tumor response assessment was performed 8–12 weeks after treatment. Morphological imaging modalities were employed (CT with contrast medium and/or MRI with or without contrast) in all patients. Using this method, the tumor response was based on the response evaluation criteria in solid tumors (RECIST) criteria (21). If feasible, the response was also assessed with functional imaging, which included (^18^F)-fluorodeoxyglucose (FDG)-PET or choline PET for prostate cancer. In this study, the European Organisation for Research and Treatment of Cancer (EORTC) criteria were used (22). Specifically, the PET-based response was assessed according to criteria including progressive metabolic disease (PMD), stable metabolic disease (SMD), a partial metabolic response (PMR) and a complete metabolic response (CMR). PMD involved an increase in the tumor (^18^F)-FDG standardized uptake value (SUV) of >25% within the tumor region defined on the baseline scan, a visible increase in the extent of tumor (^18^F)-FDG uptake of >20% in the longest dimension, or the appearance of novel tumor (^18^F)-FDG uptake in metastatic lesions. SMD comprised an increase in the tumor (^18^F)-FDG SUV of <25% or a decrease of <15%, and no visible increase in the extent of the (^18^F)-FDG tumor uptake (i.e., not >20% in the longest dimension). A partial metabolic response required a reduction of >25% in the tumor (^18^F)-FDG SUV. A reduction in the extent of the tumor (^18^F)-FDG uptake was not a pre-requesite for a PMR, whereas a CMR negated a complete resolution of the (^18^F)-FDG uptake within the tumor volume, in order that it was indistinguishable from the surrounding normal tissue. The follow-up was performed according to the scheme detailed in Table IV.

### Quality of life (QoL) evaluation

The cancer linear analog scale (CLAS) score was used to evaluate the impact of SBRS on the patient’s quality of life (CLAS1), energy level (CLAS2) and ability to undertake daily activities (CLAS3), both prior to and 3–4 weeks after radiotherapy. Patients scored their perceptions of these symptoms by placing a mark on a 100-mm line (23).

## Results

### Patient characteristics

**Figure 1:**
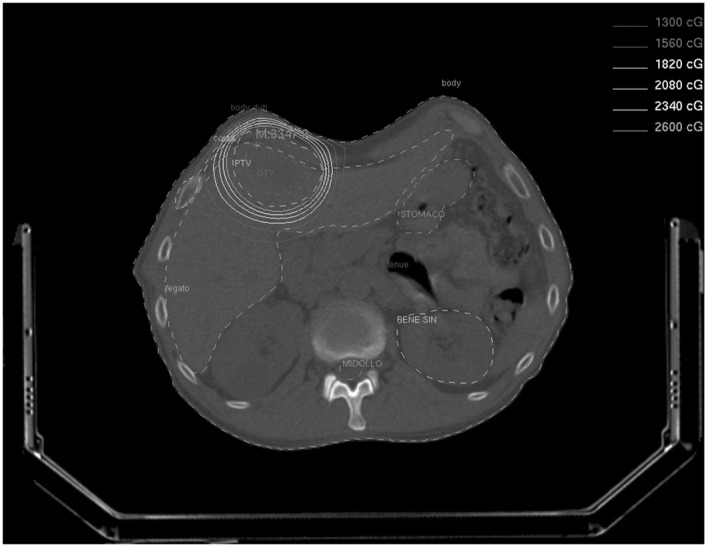
Example of dose distribution.

The preliminary analysis was based on the first 20 enrolled patients who had a total of 25 lesions (Table V). The median PTV size was 37.8 cc (range, 0.9–202.4). The prescribed dose ranged from 12–26 Gy to the PTV (Fig.1).

### Technical issues

The dose-volume constraints for OARs were observed in all patients using a single-arc technique. Only one patient, who was treated for two liver lesions, required a two-arc technique. To administer the prescribed doses, 1401.9–3246.2 monitor units (median, 2157.75) were employed with a median beam-on time of 6 min and 6 sec (range, 4 min and 0 sec to 9 min and 13 sec). In all patients, the treatment was performed without interruption or any other technical issues.

### Acute toxicity and response

**Figure 2:**
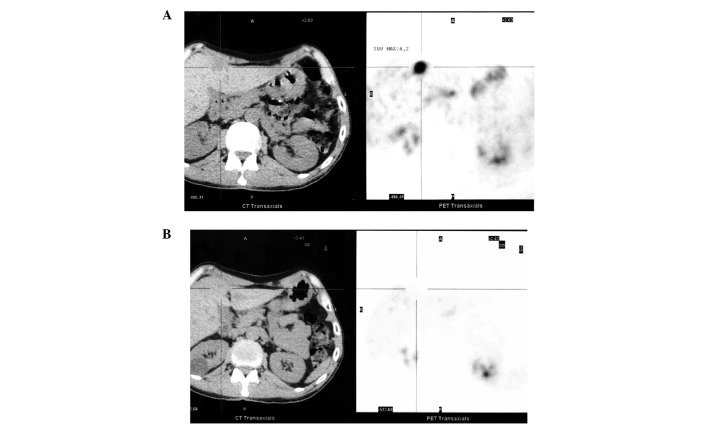
Single liver metastasis: Fluorodeoxyglucose (FDG)-positron emission tomography (PET) before (A) and after (B) radiosurgery (26 Gy).

All patients were evaluable for acute toxicity. Twenty per cent of patients experienced grade 1 acute toxicity. No patients demonstrated acute toxicity > grade 1. Twenty five lesions were evaluable for clinical response by morphological imaging. In the irradiated site, the tumor responses included 7 lesions with a complete response (CR; 28%), 5 with a partial response (PR; 20%) and 13 with stable disease (SD; 52%). Moreover, 18 lesions were evaluable for a clinical response by functional imaging as follows: 9 lesions with a CR (50%), 7 with a PR (39%) and 2 with SD (11%) (Fig. 2). No difference in the CLAS score was observed prior to SBRS compared with at the first follow-up (data not shown).

### Late toxicity and outcome

With a median follow-up time of 12 months (range, 8–20), no patients presented with late toxicity. Overall, 3 patients experienced local disease progression. One-year actuarial progression-free survival in the irradiated site was 88%, while 13 patients (65%) demonstrated progressive disease in sites different from the irradiated one.

## Discussion

We describe our initial experience with radiosurgery by VMAT. Large radiation doses were delivered to the 20 patients in this study, and the constraints of the OARs were observed and a simple single-arc technique was implemented (in 19/20 patients) in <10 min. Acute toxicity was exclusively grade 1 (CTCAE 4.03). Considering the 25 lesions, a morphological response rate of 48% (95% CI, 24.2–70.2) and a functional response rate of 89% (95% CI, 58.6–98.7) were demonstrated.

There are few studies in the literature regarding the use of stereotactic VMAT. The majority of these are dosimetric studies concerned with spine (24,25), lung (24,26–28), brain (29,30) and adrenal metastases (31). All of these studies have demonstrated an increased efficiency of VMAT in terms of treatment time, with respect to 3D-conformal or IMRT techniques. A number of these have also described improved conformity compared with 3D techniques (26–28) and a similar (30) or higher (27,28) conformity compared with standard IMRT techniques. Clinical studies are less numerous and are concerned with the spine (32–34), arteriovenous malformations (35) and abdominal targets (36). These preliminary studies have mainly documented the technical feasibility of stereotactic VMAT, and all the authors have employed this technique in fractionated treatments. To the best of our knowledge, the present series represents the first clinical study on radiosurgery using VMAT.

In terms of feasibility, we stress that the dose-volume constraints were met in all patients in the current study. The use of relatively small doses, in this first phase of the study, likely facilitated this result. In addition, the use of high doses was tolerated at least in terms of acute toxicity. Moreover, the analysis of QoL- and fatigue-related indicators prior to and following radiosurgery demonstrated that SBRS was not associated with any detrimental effects. The low number of patients and the short follow-up time mean that is is not possible to assess the local control and late toxicity. However, the high index of an immediate response, particularly if assessed with functional imaging, and the absence of relevant toxicity should be noted.

From a practical perspective, introducing VMAT for radio-surgery resulted in a marked reduction in the treatment time. In our previous experience with stereotactic radiation therapy based on non-coplanar fixed fields, a time of 45 min was reserved for each treatment. In the present study concerning VMAT, a machine time of only 20 min per treatment was reserved. Considering the promising results in terms of the feasibility and the preliminary clinical results, the study should continue with the recruitment of additional patients to the subsequent dose levels.

## Figures and Tables

**Table I:** Inclusion criteria.

Study arm	Criteria
Lung	Primary or secondary lung tumors
	Number of lesions: 1–5
	Largest diameter <5 cm
	Surgical treatment not indicated
	No prior RT at the same site
	No chemotherapy 14 days before and after SBRS
	Absence of bronchopulmonary
	Infections in active phase
Liver	Primary or secondary liver tumors
	Number of lesions ≤3 (four if two lesions <3 cm and close together)
	Largest diameter <6 cm (5 cm for 1 lesion, 4 cm for 2 lesions, and 3 cm for 3 lesions)
	Distance >6 mm from the gastrointestinal tract
	Surgical treatment not indicated
	No previous RT to the liver
	No chemotherapy 14 days before and after SBRS
	Absence of active liver infections
Bone	Bone metastases
	Number of lesions: 1–5
	Largest diameter of the single lesion <6 cm
Other	Advanced primary tumor or local recurrence or distant metastasis
	Surgical treatment not indicated
	Excluded from other arms of the study

RT, radiotherapy; SBRS, stereotactic body radiosurgery.

**Table II:** Dose levels (Gy) planned and reached (underlined) in the different arms of the study.

Level	Lung	Liver	Bone	Advanced
1	26	26	12	16
2	28	28	14	18
3	30	30	16	20
4	32	32	18	22
5	34		20	24
6			22	
7			24	

**Table III:** Dose-volume constraints.

Organ	Dose (Gy) or volume (% or cc)	Reference
Ribs	Dmax=30	NCCN v.2.2010 (18)
Heart/pericardium	Dmax=22	NCCN v.2.2010 (18)
Skin	Dmax=26	NCCN v.2.2010 (18)
Esophagus	Dmax=15.4	NCCN v.2.2010 (18)
Liver	V_12Gy_ <30%	
	V_7Gy_ <50%	Herfarth KK, 2001 (5)
Great vessels (mediastinum)	Dmax=37	NCCN v.2.2010 (18)
Bowel (small bowel/colon)	Dmax=12	Herfarth KK, 2001 (5)
Spinal cord	Dmax=14	NCCN v.2.2010 (18)
Brachial plexus	Dmax=17.5	NCCN v.2.2010 (18)
Sacral plexus	Dmax=18	Timmerman RD, 2008 (19)
Lungs	V_7.4Gy_=1000 cc	Timmerman RD, 2008 (19)
Kidneys	V_8.4Gy_=800 cc (cortical area)	
	V_10.6Gy_=2/3 volume (ilo)	Timmerman RD, 2008 (19)
Stomach	Dmax=12.4	NCCN v.2.2010 (18)
Trachea/large bronchus	Dmax=20.2	NCCN v.2.2010 (18)

NCCN, National Comprehensive Cancer Network.

**Table IV:** Follow-up.

Study arm	First follow-up	Subsequent follow-up
Lung		Chest CT and PET-CT at 3 months and every 6 months thereafter
Liver	2 weeks after SBRS to evaluate acute toxicity	Abdominal CT and PET-CT at 3 months and every 6 months thereafter; focal hepatic reaction evaluation
Bone		Bone CT and PET-CT or bone-scan at 3 months and every 6 months thereafter (anticipated if symptoms)
Advanced		Body CT and PET-CT at 3 months and every 6 months thereafter (anticipated if symptoms)

SBRS, stereotactic body radiosurgery; CT, computed tomography; PET, positron emission tomography.

**Table V:** Patients characteristics and results.

Patient	Gender	Age (years)	Study arm	Tumor	PTV (cc)	Prescribed dose (Gy)	Acute toxicity (CTCAE 4.03)	PET response	CT or MRI response	Local failure (0, no; 1, yes)	Local failure (months)
1	F	47	Bone	Breast: bone metastasis	6.2	12	0		NC	0	16
				Breast: bone metastasis	11.7	12	0		NC	0	16
2	M	65	Liver	Nasopharynx: single liver metastasis	85.4	26	Skin hyper-pigmentation G1	CR	CR	0	15
3	M	82	Advanced	Prostate: pelvic nodal metastasis	137.5	16	0	CR^a^	CR	0	13
				Prostate pelvic nodal metastasis	133.8	16	0	PR^a^	PR	1	13
4	M	59	Bone	Prostate: bone metastasis	8.1	12	0	CR^a^	CR	0	13
5	F	81	Advanced	Vagina: nodule on the right wall	75.1	16	Vaginal inflammation and pain G1	PR	NC	1	8
6	F	64	Lung	Colon: single lung metastasis	22.3	26	0		NC	0	8
7	M	67	Bone	Prostate: bone metastasis	0.9	14	0	CR^a^	NC	0	12
8	M	72	Bone	Prostate: bone metastasis	8.4	14	0	CR^a^	NC	0	6
9	M	82	Advanced	Prostate: pelvic nodal metastasis	37.8	16	0	NC^a^	NC	0	8
				Prostate: pelvic nodal metastasis	12.5	16	0	CR^a^	CR	0	8
10	F	63	Lung	Cervix: single lung metastasis	6.3	26	Pneumonitis G1, esophagitis G1	PR	NC	0	4
11	F	63	Lung	Colon: single lung metastasis	50.9	26	esophagitis G1	CR	CR	0	8
12	M	66	Bone	Prostate: bone metastasis	10.2	14	0	NC^a^	NC	0	9
13	F	70	Advanced	Breast: liver metastasis	69.4	16	0		PR	0	9
				Breast: liver metastasis	14.6	16	0		PR	0	9
14	M	56	Bone	Lung: bone metastasis	78.8	16	0	PR	NC	0	7
15	M	72	Lung	NSCLC: primary tumor	43.3	26	0	CR	CR	0	7
16	M	67	Liver	Nasopharynx: liver metastases	111.8	26	0	PR	NC	1	5
				Nasopharynx: liver metastases	52.6	26	0	PR	NC	0	7
17	F	86	Advanced	Colon: abdominal nodal metastasis	95.6	16	0	CR	NC	0	7
18	M	80	Bone	Prostate: bone metastasis	202.4	16	0		PR	0	4
19	F	67	Advanced	Cervix: single nodal metastasis	15	18	0	PR	PR	0	5
20	F	49	Liver	Colon: liver metastasis	26.8	26	0		CR	0	4

a choline-PET response. PTV, planning target volume; CTCAE, Common Toxicity Criteria for Adverse Events; PET, positron emission tomography; CT, computed tomography; MRI, magnetic resonance imaging; NSCLC, non-small cell lung cancer; CR, complete response; PR, partial response; NC, no change.
